# Telehealth Secure Solution to Provide Childhood Obesity Monitoring

**DOI:** 10.3390/s22031213

**Published:** 2022-02-05

**Authors:** Elitania Jiménez-García, Miguel Ángel Murillo-Escobar, Jesús Fontecha-Diezma, Rosa Martha López-Gutiérrez, Liliana Cardoza-Avendaño

**Affiliations:** 1 Engineering, Architecture and Design Faculty, Autonomous University of Baja California (UABC), Ensenada 22860, Baja California, Mexico; ejimenez@uabc.edu.mx (E.J.-G.); murillo.miguel@uabc.edu.mx (M.Á.M.-E.); lcardoza@uabc.edu.mx (L.C.-A.); 2 Department of Information Systems and Technologies, University of Castilla-La Mancha, 13071 Ciudad Real, Spain; jesus.fontecha@uclm.es

**Keywords:** obesity, telehealth, multiuser network, spread spectrum, chaos

## Abstract

Childhood obesity causes not only medical and psychosocial problems, it also reduces the life expectancy of the adults that they will become. On a large scale, obese adults adversely affect labor markets and the gross domestic product of countries. Monitoring the growth charts of children helps to maintain their body weight within healthy parameters according to the World Health Organization. Modern technologies allow the use of telehealth to carry out weight control programs and monitoring to verify children’s compliance with the daily recommendations for risk factors that can be promoters of obesity, such as insufficient physical activity and insufficient sleep hours. In this work, we propose a secure remote monitoring and supervision scheme of physical activity and sleep hours for the children based on telehealth, multi-user networks, chaotic encryption, and spread spectrum, which, to our knowledge, is the first attempt to consider this service for safe pediatric telemedicine. In experimental results, we adapted a recent encryption algorithm in the literature for the proposed monitoring scheme using the assessment of childhood obesity as an application case in a multi-user network to securely send and receive fictitious parameters on childhood obesity of five users through the Internet by using just one communication channel. The results show that all the monitored parameters can be transmitted securely, achieving high sensitivity against secret key, enough secret key space, high resistance against noise interference, and 4.99 Mb/sec in computational simulations. The proposed scheme can be used to monitor childhood obesity in secure telehealth application.

## 1. Introduction

Childhood obesity has increased considerably in the last decades [1,2]. The magnitude of the increase has been more than 10-fold in the last 4 decades, with a worldwide increase in the number of obese children and adolescents (5–19 years of age) from 11 million in 1975 to 124 million in 2016. Furthermore, an additional 213 million children were overweight (but not obese) in 2016 [3]. A study commissioned by the Organization for Economic Co-operation and Development (OECD) showed that childhood obesity in Mexico doubled over 20 years, from 7.5% in 1996 to 15% in 2016 [4].

The World Health Organization (WHO) defines overweigh and obesity as an abnormal or excessive accumulation of fat that poses a risk to health. For children aged 5 to 19 years, overweight is the BMI for age with more than one standard deviation above the established median in child growth patterns, and obesity is greater than two standard deviations above the median. Obesity is a difficult medical issue to overcome, and its complexity lies in its “multi-systemic nature” [5], although an inadequate diet and physical activity in childhood are suspected to be the leading causes of it [6], as well as short sleep duration that may affect energy balance [7]. Childhood obesity represents a serious health problem worldwide [8,9], as it brings about adverse health issues for those who present this condition, such as increased risks of cardiovascular diseases and diabetes, as well as psychological and gastrointestinal disorders, among other health problems. Moreover, overweight and obese individuals, including childhood obesity, can face a greater risk of health if infected with the COVID-19 virus, including hospitalization, intensive clinical care requirements, and even death [10,11,12]. It has been argued that if obesity is not controlled early in childhood, children have a high risk of becoming obese adults [13,14].

For all the reasons stated above, it is necessary to help prevent overweight and obesity in children so that the consequences mentioned above can be reduced. On the other hand, telehealth provides remote medical services by using telecommunications and data digitalization, including with computers and mobile devices, with the aim of delivering health care remotely and to manage the health of patients. Physicians can access personal medical information around the world using the Internet [15], but data privacy must be considered when insecure channels are used.

In this work, a remote secure monitoring and supervision platform is proposed that can be used in various health areas. As an example, in this article, the case of application in the prevention of childhood obesity is considered. The main contributions of this work can be summarized as follows: (1) a novel telehealth solution to monitor childhood obesity is proposed based on monitoring physical activity and hours of sleep of children based on activity bracelets, (2) a multi-user network with chaos-based data encryption is included to transmit all the parameters of children securely between parents and specialists, (3) the proposed scheme can monitor and evaluate childhood obesity and avoid personal data theft.

The remainder of this paper is organized as follows: Related work regarding with secure monitoring in telehealth is presented in Section 2. In Section 3, family-based weight control for children and the information to be sent are described based on the recommendations of published authors; in Section 4, the proposed multi-user secure communication scheme to monitor childhood obesity is presented and shows how the platform is configured. In Section 5, the experimental results of the simulations of sending and receiving fictitious data on childhood obesity with the chaotic encryption process are shown. The security analyses are presented in Section 6. Finally, the concluding remarks are presented in Section 7.

## 2. Related Work

A recent review of telehealth research showed that it is as a useful tool to manage nutrition and exercise programs for pediatric obesity in times of COVID-19. According to the results, telehealth is an effective platform to perform online nutritional support and exercise training programs to promote a healthy lifestyle in children and adolescents, including for pediatric obesity [16]. Nevertheless, security algorithms and protocols must be considered to provide privacy of personal information [17].

The properties of chaotic systems are highly related to cryptographic properties to design secure encryption algorithms, such as high sensitivity at initial conditions, mixing data, the ergodic system, the non-lineal system, and pseudorandom behavior. The spread spectrum allows multiplex code data in wireless communications to send information through a single communication channel, with robustness against noise interference [18]. The chaotic systems and spread spectrum technology have been proposed to provide security in multi-user communication systems in telemedicine, mainly focused on ensuring the privacy of biosignals such as electrocardiograms for diagnosis and disease prevention in telemedicine.

Aledhari et al. proposed a hybrid real-time cryptography encryption scheme for lightweight wearable medical devices by using genomic encryption and chaotic systems for health monitoring [19]. A chaos-based encryption process for clinical data has been proposed in [20], where one round of permutation and diffusion architecture was implemented for the privacy of electrocardiograms (ECG), electroencephalograms (EEG), and blood pressure (BP) biomedical signals. According to the security analysis, the proposed algorithm is secure for e-health applications. In 2019, Rammyaa et al. studied the performance of medical image transmission for cognitive radio multi-carrier in a code-division multiple access (CDMA) system with an image encryption algorithm based on a chaotic Arnold cat map, and the security analyses were presented [21]. In 2018, Michel-Macarty et al. presented a multi-user communication scheme based on binary phase shift keying (BPSK) and the two-dimensional Hénon map in a direct sequence spread spectrum (DS/SS) system to provide privacy of biosignals privacy and security for remote health care [22]. Basic security analyses were presented, such as key analysis for sensitivity and space key, noise interference analysis, and encryption time.

Based on the above works, we propose a telehealth secure communication scheme for monitoring parameters of childhood obesity with the aim to help in the prevention of overweight and obesity in children, which uses mainly telehealth, spread spectrum technology, and chaotic encryption in a multi-user network.

## 3. Family-Based Weight Management for Children

Childhood obesity, overweight, and their comorbidities can be prevented by carrying out continuous monitoring of children’s growth charts and documenting deviations from them [23,24,25], as an important part of a multi-faceted approach for successful treatment to reduce childhood obesity. Monitoring and evaluation are two points that can help raise awareness of the problem and are necessary to evaluate progress in the development, implementation and effectiveness of interventions. It is important to promote a healthy lifestyle early in childhood for many reasons, including to help promote a healthy weight, so as to limit the possibility of the development of obesity, as the effects of the implemented actions are likely to be long-lasting [26]. Given the high prevalence of childhood obesity and its potential deleterious, long-lasting consequences for those who present this condition, the use of telehealth solutions could provide intensive, family-based pediatric weight management that includes psychological attention, and in cases where psychological help is needed, it can be performed remotely [27].

**Figure 1:**
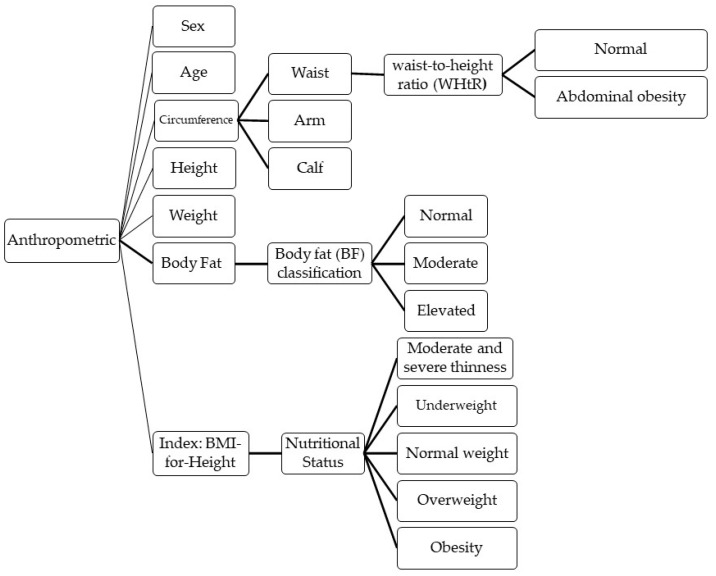
Anthropometric Data.

**Figure 2:**
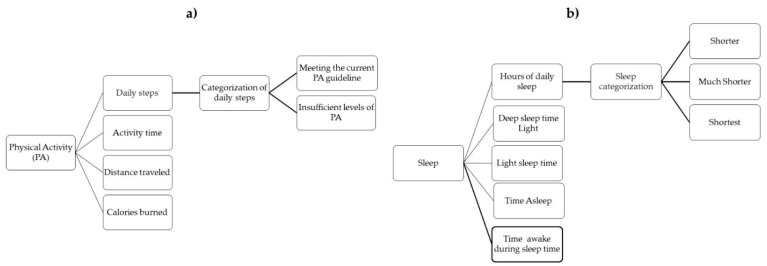
Kinds of data obtained by non-intrusive device: (**a**) Data of physical activity and (**b**) hours of sleep.

To define which risk factors contribute to the early onset of childhood obesity and overweight and which data from these will be sent by our scheme, literature reviews in the field were taken into account, seeking further categorization, as well as cut-off points for some parameters that determine the risk or prevalence of childhood obesity and overweight. The chosen parameters were anthropometric data, physical activity, and hours of sleep. However, the system can be extended to more categories of data that are also valuable in determining the risk of childhood obesity, such as diet, which was not included at this stage due the difficulties in the accurate assessment of dietary intake. The data and their categorizations that are to be sent to health professionals are shown in Figure 1 and Figure 2a,b.

Body measurements (waist, arm, and calf circumference) are taken by the parents, for which they will receive a basic manual with simple instructions. This manual will be based on the recommendations of recognized health organizations in the region where the proposed scheme will be implemented. Additionally, the parent will weigh the child, and enter the data into an application on their cell phone. From the previous data, the mobile application will first validate the data entered by the parent to verify that there are no significant variations with previous data (if any). If so, it will inform the parent to rectify the measurements. With the data provided, the mobile application will calculate the waist-to-height ratio (WHtR) and the body mass index (BMI) and send this data to the health professionals through the platform. It should be mentioned that three key anthropometric indicators of childhood obesity and overweight were considered: Two of them can be obtained from the body measurements (WHtR and BMI) and the third, body fat, will be used only when the parents have the digital scales to obtain these data. Although the accuracy of digital scales (or smart scales) for estimating body composition, including body fat, is poor, a recent study found that such a method to obtain body fat allows the participants significant weight loss for several months [28].

For the WHtR, which is used to determine the prevalence of abdominal obesity, the cut-off point considered as the boundary value to assess risk is 0.5 [24,25]. This indicator is recommended for the evaluation and follow-up of obese and overweight children [26]. For the second indicator, body fat, the cutoffs for the percentage of body fat were chosen so that within each sex-age group, the number of children in the fatness categories (normal, moderate, and elevated) would be equal to that in the corresponding body mass index for age categories (normal weight, overweight, and obese, respectively) [29]. For the last indicator, BMI, which is a simple index of weight-for-height that is commonly used to classify overweight and obesity, we are using the WHO growth reference data.

In addition to the growth patterns, the children will wear an activity bracelet which will be linked to the parents’ mobile phone. The bracelet will collect information on physical activity and sleep hours (sum of light and deep sleep time) of the children and will synchronize that information with the parent’s smart phone and a mobile application will access and select the data to be sent securely to health professionals by using the Internet. These data and their categorizations are shown in Figure 2. Light sleep time is considered when breathing slows down, the muscles relax, and the brain waves are less active, whereas deep sleep time light is considered as the short time when breathing, heartbeat, body temperature, and brain waves reach their lowest levels and the body repairs tissue and cellular energy is restored.

Detection of insufficient levels of physical activity and insufficient sleep hours is essential for the identification of children and adolescents more prone to develop obesity [30], although it is difficult to accurately measure these habits. Subjective measures, such as self-reported questionnaires, are compromised by the inability of children and parents to accurately remember all relevant details about their daily patterns of physical activity, the level of intensity of physical activity, and their daily sleep hours. For that reason, we are proposing to collect both physical activity and sleep by using basic activity bracelets.

Physical inactivity is undoubtedly one of the factors that has a significant impact on childhood obesity. According to international recommendations, children from 5 to 17 years of age should perform at least an average of 60 minutes of moderate to vigorous physical activity (MVPA) daily [31]. We propose the use of bracelets to objectively measure the child’s physical activity and express it as absolute counts and minutes dedicated to an activity of a particular intensity. Da Silva et al. has suggested that the most optimal step count for children and adolescents (5–19 years) is 12,000 steps per day, which indicates that the physical activity recommendations are being met [32].

For the number of sleep hours, after conducting a systematic review, Morrissey et al. [33] found that there is an association between short sleep duration and less favorable diet quality in childhood. Short sleepers tend to select foods with higher energy density, more added sugar, and drinks sweetened with sugar than long sleepers [34,35]. In their systematic review, Miller et al. found that the short duration of sleep is a risk factor for the development of obesity in infants, children, and adolescents [36].

For the classification of sleep duration and the recommended duration of sleep hours, we used those reported by Chen et al., where the standardized sleep duration reference was defined as shown in Table 1 [35].

## 4. Proposed Multi-User Secure Monitoring Scheme

**Figure 3:**
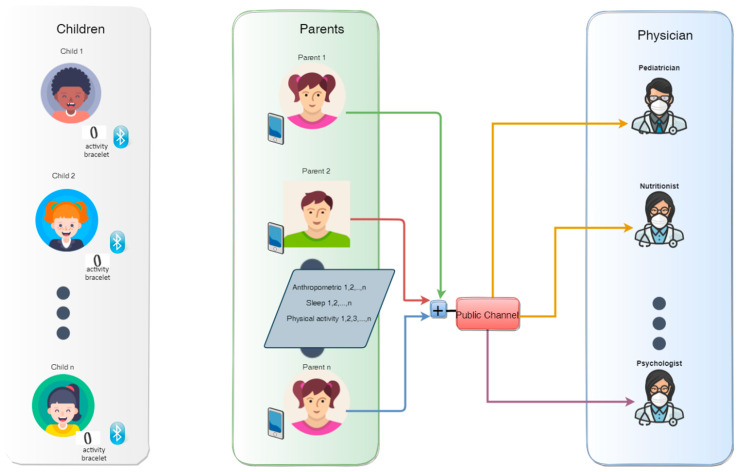
Architecture of the proposed transmission of medical information in a multi-user network.

We propose a telehealth scheme that allows for the remote monitoring of the risk factors that contribute to the potential development of childhood obesity and overweight. The data will be collected by parents and by means of non-intrusive electronic devices, such as a smart phone, to process the data and send them securely through the communication system to health professionals. The specialist will be able to evaluate the level of obesity, if present, or its risk of occurrence in children and adolescents (6 years and older), and provide parents with clear, accurate, and meaningful information and recommend behavioral interventions that promote a better weight status for the children. The data obtained from the children by means of an activity bracelet (non-intrusive devices) are synchronized with the parents’ smartphone. The system developed for this scheme, will show a weekly report to the parent, in addition to transferring such data to the physician every 6 months or in shorter periods of time, depending on the degree of obesity of the child, and as established by the health professionals. In the proposed telehealth monitoring system, multi-user encryption uses chaotic system that are combined with the code-division multiple access (CDMA) technique and spread spectrum technology to provide data privacy and transmission by using just one communication channel (Internet as insecure public channel) between parents and physicians. The general scheme of the architecture of telehealth is shown in Figure 3.

First, children’s physical activity and sleep hours will be monitored daily using activity bracelets. When this information on risk factors is outside the recommended ranges, a warning will be sent to the parent’s smart phone by using a Bluetooth communication protocol to invite them to motivate their children to comply with the recommendations. Then, parents, in agreement with their health professionals, will take frequently take the anthropometric measurements of their children, which will in turn be sent to the health professionals using the Internet (4G, 5G, or Wi-Fi protocol), with the encrypted weekly report of physical activity and sleep hours, in addition to the categorization of the data as shown in Figure 1 and Figure 2. Telemedicine is a valuable technology that link clinicians with patients to ensure they make long-term lifestyle changes, it could be making health more affordable reducing distance limitations by exchanging information about a diagnosis, healthcare, and disease prevention between the doctor and the patient through electronic means [15], and for this reason, it is important to assure data integrity.

The encryption algorithm of Michel-Macarty et al. [22], which is based on the chaos and spread spectrum technique with symmetric-key cryptography, is used as a reference to provide information confidentiality in the proposed communication scheme. The encryption algorithm is briefly transcribed and adapted to the propose communication scheme as follows:

1. The secret key is defined for each child-parent-specialist in a symmetric-key cryptography.
2. The plain text is defined as a vector of 10 × 16-bit unsigned integer for *N* users. The binary plain text is coded to non-return zero (NRZ), i.e., 1 for “1” and −1 for “0”.
3. The NRZ plain text of each user is modulated with binary phase shift key (BPSK) using a carried signal defined as cosine signal at 1 Hz, 1 amplitude, and 50 samples per cycle.
4. The chaotic two-dimensional Hénon map is used for each user, which is defined as follows:
  (1) $$\begin{array}{c} x_{n+1}=1-ax_{n}^{2}+y_{n},\\ y_{n+1}=bx_{n}, \end{array}$$
  where a and b are two control parameters, and $x_{0}$ and $y_{0}$ are two initial conditions, which are considered as the secret key of each user. Digital words of 64-bit floating-point are used for the Hénon map to generate 15 decimals. Chaotic data are amplified by 1,000 and the module 1 operation is used to increase chaotic uniformity. The chaotic data (*CD*) with values of (0,1) are coded to NRZ based on the following criteria:
  (2) $$CDNRZ=\{\begin{array}{cc} 1 & if\;CD\geq 0.5,\\ -1 & Otherwise, \end{array}$$
  where *CDNRZ* is the chaotic data coded in NRZ.
5. The plain text modulated with BPSK and the chaotic data coded in NZR are multiplied to produce the spread spectrum signal of each user. Finally, all spread spectrum signals of *N* users are summed to produce the cryptogram.

The algorithm to retrieve the plain text from the cryptogram is transcribed as follows:

1. The authorized specialist receives the cryptogram.
2. With the corresponding secret key of the user *N*, the authorized specialist uses the same chaotic Hénon map and generates the *CDNRZ* as in encryption process.
3. The same carried signal defined in the encryption process is generated.
4. The cryptogram is multiplied with *CDNRZ* to generate the inverse spread spectrum signal.
5. The sign function with a length of 50 samples is applied to the inverse spread spectrum to retrieve the plain text in binary format according to the following criteria:
  (3) $$PTB=\{\begin{array}{cc} 1 & if\;sng\left(\;\right)>0,\\ 0 & Otherwise, \end{array}$$
  where *PTB* is the recover plain text in binary format.
6. Finally, groups of 16-bit data are converted to decimal format to retrieve the original plain text of the *N* user.

## 5. Experimental Results

The proposed telehealth secure communication scheme for childhood obesity monitoring was implemented using MATLAB R2015a software [37]. The hardware used was a laptop computer with Intel CORE i3 CPU 2.0 GHz, 64-bit windows 10 operating system, and 8 GB of RAM. The main objectives of this implementation were to transmit childhood obesity parameters securely by using the architecture in Figure 3 and to determine its level of security and efficiency using the MATLAB software environment. We assumed that parents had collected the data previously using smartphones and Bluetooth communication with the activity bracelets used by their children.

In experimental results, we considered a simulated scenario based on five children (five users), with plain text fictitious data related to their health, such as exercise and sleep (Table 2). Such fictitious data are similar to real cases as presented in data sets in [38]. We considered that children used an activity bracelet to measure the data. Then, the results are based on the evaluation of the proposed multi-user secure communication for secure telehealth monitoring. The experimental results are limited to children’s weekly exercise and sleep data. The times of the Fell asleep at and Woke up parameters are converted to a string of just numbers, where the hour is multiplied by 60 plus the minutes, e.g., 8:08 Hrs. are converted to 488, as seen in Table 2 for the Woke up parameter of User 1.

For the encryption process, the secret key for each user is defined in Table 3. There are two initial conditions and two control parameters for each Hénon map and user. Since the 64-bit double floating-point format is used, each value has 15 decimal precisions. Since all of the encrypted data of the children are transmitted together to the physician by using just one communication channel, the proposed scheme can reduce the cost of implementation.

**Figure 4:**
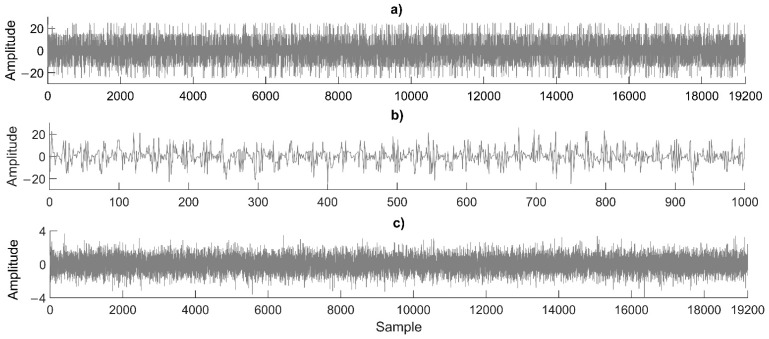
Cryptogram: (**a**) complete cryptogram with noise, (**b**) first 1,000 data points of cryptogram, and (**c**) AWGN noise added.

The cryptogram generated is shown in Figure 4. Figure 4a presents the cryptogram signal (with additive white Gaussian noise (AWGN) and a signal-to-noise ratio (SNR) of 1 dB based on MATLAB [37]) to be transmitted over an unsecured channel with 19,200 data points (64-bit double floating-point format IEEE 754 standard [39]). Figure 4b shows the first 1000 data points of the cryptogram. In Figure 4c, the AWGN noise added to the cryptogram is shown.

The cryptogram is sent by the parents over an unsecured channel, such as the Internet, to the corresponding physician or specialist. The encryption algorithm is considered public, but the secret key must be confidential between the authorized parties (parents and physicians). An intruder could acquire the cryptogram from the Internet but only the authorized receiver who has the correct secret key can recover the plain text or the weekly monitored data of the children.

The secret keys to recover the plain text of the five users (children) are shown in Table 4. Note that only three keys are correct (users 2, 3, and 5), whereas the other two are incorrect in the last digit in $x_{0}$ and $y_{0}$, respectively (users 1 and 4). The recovered plain text of the five users is shown in Table 5. The plain text cannot be recovered correctly for users 1 and 4, whereas the plain text is recovered correctly for users 2, 3, and 5, even with noise added in the cryptogram.

Therefore, the plain text can be recovered by the physician even if the transmitted cryptogram has noise interference. This is due to the advantages of using spread spectrum technology and codification based on chaos. Haleem et al. present schemes for monitoring childhood obesity [15], but these do not consider data security.

## 6. Security Analysis

In this section, the security analyses are presented to show the effectiveness of the proposed implementation. Analyses such as secret key space, secret key sensitivity, robustness against noise attack with bit error rate, and encryption time are described.

### 6.1. Key Space Analysis

An exhaustive search attack could be implemented by using super computers, where each possible key is used until the correct plain text is retrieved. The key space must be at least $2^{128}$. to resist this attack. In the proposed scheme, each secret key is based on two control parameters and two initial conditions (a, b, $x_{0}$, and $y_{0}$), with 64-bit floating-point arithmetic (IEEE 754 standard [39]), i.e., with 15 decimal precision for each number, the key space is $10^{15}+10^{15}+10^{15}+10^{15}\approx 2^{199}$.

Thus, the key space is enough to resist an exhaustive search attack using super computers. In addition, the floating-point computation under 64-bit double precision allows a huge key space and avoids digital degradation of chaos.

### 6.2. Key Sensitivity Analysis

The ability to produce two uncorrelated cryptograms using two highly similar secret keys must be performed in any encryption algorithm, which is called the avalanche effect. The use of chaotic maps helps in this ability of the encryption algorithm, since they are highly sensitive to the initial conditions and control parameters. In the decryption process, only the correct secret key must recover the original plain text. Table 5 depicts the key sensitivity results for the decryption process considering the secret keys in Table 3. The plain text is recovered correctly for users 2, 3, and 5 (since the same secret key of encryption is used), whereas users 1 and 4 cannot recover correctly the plain text (since secret keys are lightly different than the encryption keys).

The numerical difference between the plain text of the five users and the corresponding decrypted text is calculated using the bit error rate (BER) with the “*biterr*” function of MATLAB [37]. The BER ratio is 47.91, 0, 0, 50.78, and 0, for users 1, 2, 3, 4, and 5, respectively. The results show that the decrypted text (for users 1 and 4) has close to 50% of different bits.

Hence, the proposed telehealth monitoring scheme presents high sensitivity to the secret key in the decryption process, even when the last digit in the secret key is slightly different from the correct secret key. In comparison, the implementation in [22] found BER ratios with values of 0.5677 and 0.6543 when incorrect secret keys were used in the decryption process.

### 6.3. Bit Error Rate

The cryptogram can be altered either by noise attack or noise jamming during the transmission process, which can affect the ability to correctly recover the original plain text in the receptor. The noise robustness is presented with BER in percentage between the plain text and the recovered (decrypted) text. The results are based on different scenarios of additive white Gaussian noise (AWGN) added to the cryptogram of the five users, with a signal-to-noise (SNR) ratio of 0.001 dB, 0.01 dB, 0.1 dB, 1 dB, 3 dB, and 0 dB. The lower the SNR, the greater the noise added. Correct and incorrect secret keys in Table 4 are used, where only users 2, 3, and 5 use the correct secret key. In this sense, the recovered text of users 1 and 4 is totally different to the (original) plain text, as can be seen in Table 5. The BER is calculated with the “*biterr*” function of MATLAB [37], and the results are presented numerically in Table 6. Users 1 and 4 have a BER ratio (in percentage) close to 50% for all cases with any SNR added noise, which indicates that decrypted text and plain text are very different, since incorrect secret keys were used in all noise cases. Conversely, the BER ratio for users 2, 3, and 5 is less than 1% in the worst case of AWGN noise (0.001 SNR). The BER ratio of 0% means that the decrypted text is identical to the plain text.

Based on the above results, the proposed scheme is highly robust against noise attack or noise jamming, since less than 1% of bits are lost despite high levels of noise added to the cryptogram. Considering that each user has plain data of 160 bits, only 1 bit was lost in the decryption process.

### 6.4. Encryption Time and Throughput

With the use of MATLAB programming, and using the hardware described in Section 4 (Experimental results), the functions “tic” and “toc” are used, considering five users with 384 bits per user and the algorithm [22]. The cryptogram has 19,200 data points, with 64-bit double floating-point format, i.e., 1,228,800 bits. The average encryption time is 0.246374 s, and the average decryption time is 0.211884 s. The throughput can be calculated as the total transmitted bits over the elapsed encryption time, thus the throughput is 4.99 Mb/sec. In [22], the throughput in a three-patient scenario with four biosignals is 0.66 Mbps.

## 7. Conclusions

Childhood obesity is a serious problem worldwide, with serious adverse consequences for those who present this condition. The remote monitoring of children’s weight can be very helpful to ensure that they comply with the daily recommendations for key factors that can be promoters of obesity. Hence, the importance of developing remote monitoring to successfully carry out this effort on a large scale and in a timely manner. Telehealth appears to be useful solution. The advantage is the continuous monitoring of children. For this purpose, a telehealth monitoring system has been proposed that consists of three parts: (1) collecting data from children and monitoring their obesity-promoting parameters, (2) informing parents and physicians about the anthropometric indicators and about whether children comply with the daily physical activity and sleep recommendations, and (3) finally, health professionals provide parents with clear, accurate, and meaningful information and recommend behavioral interventions that promote improvement in children’s weight status. Due to the sensitivity of the information sent through public channels, it is essential to safeguard the information from the monitoring of children. Therefore, such information is encrypted with methods that ensure the confidentiality of the data. The information is transmitted simultaneously through the public channel. The encryption system is based on a multiuser network, spread spectrum, and chaotic Hénon map, which is capable of encrypting/decrypting the information of each child and their monitoring parameters. Based on simulation results, high security, high resistance against noise interference, and efficiency in the encryption process were achieved with high sensitivity against the secret key, enough space key to resist exhaustive search attack, a BER of zero under 0.001 SNR, and achieving a speed of 4.99 Mb/s. Another advantage of the system is its flexibility to be extended to *n* children with *m* monitoring parameters and *k* specialists. In future work, the physical implementation and its evaluation of scalability and secure data transfer over various numbers of patients will be considered.

## Figures and Tables

**Table 1:** Recommended sleep duration hours.

Age Group (years)	Recommended Sleep (hours)
<5	>11
5–10	>10
>10	>9

**Table 2:** Plain text fictional data of 5 users (children).

	User 1	User 2	User 3	User 4	User 5
Weekly steps (mean)	12,891	10,616	12,221	10,829	14,504
Weekly activity (mean min)	160	134	154	144	184
Weekly distance (km)	47	30	38	31	44
Calories burned (cal)	1208	700	1039	745	1023
Sleep (mean min)	533	480	562	511	546
Deep sleep (mean min)	136	217	220	149	146
Light sleep (mean min)	396	262	342	181	400
Fell asleep at	675	326	630	657	651
Woke up at	488	447	472	465	481
Awake time (mean min)	0	0	0	16	2

**Table 3:** Secret key definition for encryption process of the five users.

	a	b	$x_{0}$	$y_{0}$
User 1	1.400112233445566	0.300112233445566	0.556677889900112	0.667788990011223
User 2	1.400223344556677	0.300223344556677	0.667788990011223	0.778899001122334
User 3	1.400334455667788	0.300223344556677	0.778899001122334	0.889900112233445
User 4	1.400445566778899	0.300334455667788	0.889900112233445	0.990011223344556
User 5	1.400556677889900	0.300445566778899	0.990011223344556	0.001122334455667

**Table 4:** Secret key definition for the decryption process of the five users.

	a	b	$x_{0}$	$y_{0}$
User 1	1.400112233445566	0.300112233445566	0.556677889900118	0.667788990011223
User 2	1.400223344556677	0.300223344556677	0.667788990011223	0.778899001122334
User 3	1.400334455667788	0.300223344556677	0.778899001122334	0.889900112233445
User 4	1.400445566778899	0.300334455667788	0.889900112233445	0.990011223344557
User 5	1.400556677889900	0.300445566778899	0.990011223344556	0.001122334455667

**Table 5:** Recovered plain text of five users, where bold numbers denote incorrect data.

	User 1	User 2	User 3	User 4	User 5
Weekly steps (mean)	**4801**	10,616	12,221	**5416**	14,504
Weekly activity (mean min)	**11,734**	134	154	**44,277**	184
Distance week (km)	**818**	30	38	**33,968**	44
Calories burned (cal)	**32,953**	700	1039	**482**	1023
Sleep (mean min)	**56,196**	480	562	**19,737**	546
Deep sleep (mean min)	**36,955**	217	220	**61,087**	146
Light sleep (mean min)	**44,513**	262	342	**15,880**	400
Fell asleep at	**6900**	326	630	**56,226**	651
Woke up at	**22,135**	447	472	**12,731**	481
Awake time (mean min)	**23,445**	0	0	**58,027**	2

**Table 6:** BER between plain text and decrypted text for noise robustness.

SNR (dB)	0.001	0.01	0.1	1	3	0
User 1 BER (%)	45.05	53.12	47.91	48.95	48.95	48.69
User 2 BER (%)	0.89	0.59	0. 59	0.59	0	0
User 3 BER (%)	0.89	0.29	0.19	0.29	0.29	0
User 4 BER (%)	50.52	48.17	50.00	51.56	49.47	50.78
User 5 BER (%)	0.59	0.52	0.59	0.27	0.26	0
